# The Provision of Education and Employment Support At the Outreach and Support in South London (OASIS) Service for People at Clinical High Risk for Psychosis

**DOI:** 10.3389/fpsyt.2019.00799

**Published:** 2019-11-08

**Authors:** Stefania Tognin, Lara Grady, Serena Ventura, Lucia Valmaggia, Victoria Sear, Philip McGuire, Paolo Fusar-Poli, Tom J. Spencer

**Affiliations:** ^1^Department of Psychosis Studies, Institute of Psychiatry, Psychology and Neuroscience, King’s College London, London, United Kingdom; ^2^Outreach and Support in South London (OASIS) Service, South London and Maudsley NHS Foundation Trust, London, United Kingdom; ^3^Department of Psychological Medicine, Institute of Psychiatry, Psychology and Neuroscience, King’s College London, London, United Kingdom; ^4^Early Psychosis: Interventions and Clinical-detection (EPIC) Lab, Department of Psychosis Studies, Institute of Psychiatry, Psychology and Neuroscience, King’s College London, London, United Kingdom; ^5^Department of Brain and Behavioral Sciences, University of Pavia, Pavia, Italy; ^6^Maudsley Biomedical Research Centre, National Institute for Health Research, South London and Maudsley NHS Foundation Trust, London, United Kingdom

**Keywords:** education, employment, vocational support, clinical high risk for psychosis, early detection in psychosis

## Abstract

Clinical services for the early detection of individuals at clinical high risk of psychosis, such as Outreach and Support in South-London (OASIS), have been successful in providing psychological intervention and psychosocial support to young people experiencing emerging signs of serious mental disorders. Despite this, several studies have repeatedly shown that vocational and functional recovery in the clinical high risk for psychosis population is still low. This study aimed at evaluating the presence and nature of educational and employment focused interventions within the OASIS service, in order to inform research and clinical interventions aimed at supporting young people with early signs of psychosis on their path to vocational recovery. The specific objectives were to compare current practice i) to standards defined by the National Institute of Care Excellence guidelines; and ii) to principles defined by Individual Placement and Support (IPS). Nine standards of practice were derived. The OASIS caseload electronic records entered between January 2015 and January 2017 were manually screened. Data collected include sociodemographic, assessment of employment and educational status and support needs, interventions received, contacts with schools, employers and external vocational providers, employment, and educational status. Standards were considered as “met” if they were met for at least 90% of clients. Results suggest that, two out of nine standards were met while the remaining standards were only partially met. In particular, support provided was always focused on competitive employment and mainstream education and support was always based on people’s interest. Implications for clinical and research practice are discussed.

## Introduction

The first symptoms of psychosis typically emerge around late adolescence and early adulthood (1), a time during which a young person is devoting full-time to complete compulsory education or is about to enter the job market. At their first contact with early detection for psychosis services (EDP), many young people who meet criteria for being at ultra high risk of psychosis [UHR (2)], more broadly termed as clinical high risk for psychosis [CHR-P, hereafter (3)] are already falling out of education or are experiencing difficulties in finding or keeping an employment (4). In fact, they display functional impairments of a level that is comparable to that of other established mental disorders (5). Decline in social and occupational functioning often continues despite the regular contact with clinical services (4, 6, 7) and is a core predictive factors of poor clinical outcomes (8). As a result of this, CHR-P individuals often either do not complete their studies or they do so without reaching their full potential (9) with consequent future difficulties in securing competitive employment (4).

The rates of unemployment in CHR-P individuals at the time in which they first access EDP services are high. About one third of CHR-P individuals are unemployed or are not in education, and this figure is similar across different clinical services (4, 6–8). When looking at the short to medium-term outcome, despite some differences across countries, rates of unemployment—excluding students—range between 25 and 40% (4, 6, 7, 10). This indicates that despite specialized treatment being offered early, the type of interventions currently available might not be effective in preventing or improving social and occupational functioning decline and it is in line with persistent symptoms and disability in a substantial proportion of these clients (11).

To date, the evidence base for psychosocial interventions for CHR-P individuals mainly involves cognitive behavioral approaches or family intervention that aim at targeting symptoms reduction as opposed to overall social and occupational functioning (12). In line with this, several studies have repeatedly shown that vocational and functional recovery in CHR-P samples is still dramatically low (6, 8, 13, 14) and when low functioning is present at intake this is often predictive of worse long-term outcome (8, 10, 13, 15). Thus, there is an urgent need for improving social and occupational functioning recovery in this population.

For young people with emerging psychosis employment and education are highly desired outcomes and are often prioritized over relationship, housing, and symptom reduction (16). Despite this, young people with emerging psychosis are often at a disadvantage with regards to participating in education or employment which, amongst other factors, is also due to low expectations and fears of health care professionals (17, 18).

The recent National Audit for Schizophrenia (19) highlighted substantial variations in service delivery of vocational support with over half of service users not having their vocational needs met. The “Early Intervention in Psychosis Access and Waiting Time Standards” (20) state that mental health services should assist CHR-P individuals to engage with employment, education or training. However, to date, there is no indication about which intervention should routinely be employed. To add further complexity to the picture, young CHR-P individuals are a clinically heterogeneous group and are therefore more likely to require individualized treatment (21, 22).

IPS is the most successful evidence-based intervention developed to support individuals with severe mental illness gaining competitive employment (23–25). This intervention has been tested with people with a first episode psychosis (26–28) and has recently been expanded to also target educational outcomes in the same population (27, 29). However, to date, there are no randomized controlled trials investigating the benefits of vocational interventions within EDP services. Individualized interventions targeting education and/or employment, such as IPS, could significantly improve functioning, an area which has not been the primary focus of current CHR-P treatments (30). Young people accessing EDP services could be the ideal target group for this intervention for at least three reasons: i) CHR-P individuals are young and likely to be in education or to be in the process of securing their first paid job; ii) compared to patients who have experienced a first episode of psychosis, CHR-P individuals are experiencing less severe cognitive and clinical symptoms (31), iii) IPS can address key risk factors, such as unemployment and low educational level, that impact their clinical outcomes (15). In addition, some of the IPS principles, such as focusing on competitive as opposed to supported employment; attention to client preferences; benefit counseling (32) would likely already be in use in the EDP teams.

The aim of this study was therefore to evaluate the presence and quality of educational and employment focused interventions in Outreach and Support In South London (OASIS), a clinical service for CHR-P individuals within the South London and Maudsley (SLaM) NHS Foundation Trust (33) to inform clinical practice and future research in this area. The specific objectives were to compare current practice around the provision of education and employment support to i) standards defined by the National Institute of Care Excellence guidelines (34, 35); and ii) principles defined by IPS (24).

## Methods

OASIS services are part of SLAM and currently cover four boroughs: Lambeth, Southwark, Croydon, and Lewisham. OASIS was established in 2001 and receive on average about 300 referrals each year, one third of each will eventually meet criteria for a CHR for psychosis state (33). Data presented in this work were collected as part of a clinical audit which received approval from the Psychosis Clinical Academic Group in February 2018. Clinical electronic records for all clients who were accepted into the OASIS service between January 2015 and January 2017 were screened between March and November 2018. All clients meet criteria for an at risk mental state for psychosis as defined by the Comprehensive Assessment of an At Risk Mental State (2). As NICE standards partially overlap with IPS principles, nine standards based on the NICE guidelines for adults (33) and children and young people (34) with psychosis and schizophrenia and on IPS principles were developed (see **Table 1** and below). NICE guidelines are widely recognized for their high standard and the wide body of evidence they draw upon and they form the basis for many clinical audits (36). Employment and education status at intake were collected by reviewing intake forms or/and care plans. At follow-up, these were collected by reviewing notes or/and discharge letters. In order to assess adherence to the nine standards, clinicians’ notes, care plans, and outcome measurements were comprehensively screened by two experienced clinical researchers supervised by two senior clinicians. Clear evidence in clinical notes, correspondence, or care plan was necessary in order to code information, unclear evidence was conservatively considered as “not available information”. Data were included if an individual had been under the care of OASIS for at least 6 months. Based on previous studies, a minimum of 6 months was deemed sufficient to allow vocational assessment and intervention (28, 29). Anonymized data was analyzed using IBM SPSS version 24. Demographic data was analyzed using means and standard deviations for continuous data and frequencies for categorical data. As OASIS teams in Croydon and Lewisham were set up in 2014–2015 while those in Southwark and Lambeth were established in 2001, we also compared how the standards were met across all four OASIS SLaM boroughs (Lewisham, Croydon, Lambeth and Southwark) using χ2.

**Table 1:** Standards.

Standard 1	Quality of vocational assessmenta. Assessment of current vocational engagement Assessment of vocational goals Assessment of vocational support needs Assessment of vocational history
Standard 2	Vocational activities feature in care plan
	Expected: all clients
Standard 3	Clients have access to a vocational support program
	Expected: all clients (when appropriate)
Standard 4	Early psychosis services liaise with educational and employment providers
	Expected: all clients (when appropriate)
Standard 5	Early psychosis services liaise with local stakeholders
	Expected: all clients (when appropriate)
Standard 6	Support is focused around competitive employment/mainstream education
	Expected: all clients (when appropriate)
Standard 7	Support is provided based on people’s interest
	Expected: all clients (when support is provided)
Standard 8	Support is time unlimited^1^
	Expected: all clients (when support is provided)
Standard 9	Benefits counseling is provided
	Expected: all clients (when appropriate)

^1^Support is provided for as long as the client is under the care of the team and as long as support is wanted.

### Definition of Standards

#### Standard 1: Quality of Vocational Assessment

Standard 1 relates to quality of vocational assessment and is divided into (1.a) assessment of current vocational engagement; (1.b) assessment of vocational goals; (1.c) assessment of support needs; and (1.d) assessment of vocational history. Standard (1.a) is based on the NICE guidelines (35) for adults with psychosis and schizophrenia, section 1.3.3.1 and on the NICE (34) guidelines for children and young people with psychosis and schizophrenia, section 1.3.4. The guidelines do not specifically state to additionally assess vocational goals (1.b), support needs (1.c), and history (1.d) however NICE guidelines state that services should provide vocational interventions (34: section 1.5.8.1; 33: section 1.1.5 and section 1.3.9) and one of the foundational principles of supporting people with vocational engagement and goals is the detailed assessment of their previous experiences, their wishes, and their support needs (37).

#### Standard 2: Vocational Activities Feature in Care Plan

Standard 2 relates to the formal recording of vocational activities within the care plan and was based on a key recommendation within the NICE guidelines for adults (35) as stated in section 1.5.8.3 and within the NICE guidelines for children and adolescents (34) as stated in section 1.3.6. According to SLaM Care Programme Approach Policy, the care plan needs to be developed in collaboration with the service user. Therefore, the formal recording of vocational activities is a further indicator that these have been assessed and discussed with the client.

#### Standard 3: Clients Have Access to a Vocational Support Program

Standard 3 is based on the NICE guidelines (35), section 1.5.8.1, which recommend supported employment program, including support around educational activities. The NICE guidelines for children and adolescents (34) recommend support for young people to continue their education in section 1.3.4 or facilitate alternative input for people who are currently unable to attend mainstream schooling, as stated in section 1.3.9. As there was no vocational specialist within the OASIS teams during the period the audit took place, clients were not specifically referred to a vocational program within the team. Instead, support was often provided during discussions with psychologists and care coordinators or *via* referral to external program. For the purpose of the audit the definition of vocational support was kept broad and included any form of intervention aimed at supporting clients with their vocational goals and needs. Examples of support are writing supporting letters to educational institutions, longer-term psychological support aimed at managing, for example, anxiety in the work place.

#### Standard 4: Early Psychosis Services Liaise With Educational and Employment Providers

Standard 4 is based on the NICE guidelines (34) on psychosis and schizophrenia in children and young people, which recommend for early psychosis services to liaise with educational providers (section 1.1.5). It is also influenced by one of the IPS principles which requires the clinical team to liaise and build relationships with employers (32).

#### Standard 5: Early Psychosis Services Liaise With Local Stakeholders

Standard 5 is based on section 1.5.8.2 of the NICE guidelines (35) and section 1.8.14 of the NICE (34) which highlight the value of including a local and diverse range of stakeholders in the process of supporting people with their vocational needs. Additionally, the NICE guidelines (34) recommend to jointly work with people’s parents or careers (sections 1.3.6 and 1.8.12).

#### Standard 6: Support Is Focused Around Competitive Employment or Mainstream Education

Standard 6 is not specifically drawn from early psychosis guidelines although the NICE guidelines (34) on psychosis and schizophrenia in children and young people mention educational support aimed at mainstream education. This standard is a key principle of IPS (32) which recommends supporting people into mainstream employment and education as opposed to special program or voluntary work.

#### Standard 7: Support Is Provided Based on People’s Interest

Standard 7 is not stated in the NICE guidelines (2014, 2013) but was included as it is a key principle within the IPS model (32) which recommends that services are based on clients’ preferences and choices rather than providers’ judgment.

#### Standard 8: Support Is Time Unlimited

Standard 8 is not stated in the NICE guidelines (34, 35) but was included as it is a key principle within the IPS model (32) which recommends that support is provided for as long as the client wants and needs the support.

#### Standard 9: Benefits Counseling Is Provided

Standard 9 was included as it is a key principle within IPS (32). In the UK, this would translate, for example, into giving advice on permitted work hours when in receipt of state benefits such as the Employment and Support Allowance, changes to housing benefits, if applicable, and accessing student loans and grants as well as grants for business start-ups.

### Coding of Standards

Standards 1a, 1b, 1c, 1d, and 3 were coded as follows: “no,” “at initial assessment” (standard 1 only); “within 3 months”; “within 6 months”; “within 12 months”; “after 12 months.” Standard 2 was coded as follows: “yes”; “no”; “no care plan.” Standard 4 was coded as follows: “yes”; “no”; “not employed/not in education”; “employer/school/university already aware”; “client liaised with employer/school/university.” Standard 5 was coded as follows: “parents/relatives”; “external stakeholders”; “job centre”; “recovery college”; “other clinicians”; “multiple stakeholders”; “others”; “no stakeholders involved.” Standards 6, 7, and 9 were coded as follows: “yes”; “no”; “not applicable.” Standard 8 was coded as follows: “ongoing”; “none provided”; “termination of psychology sessions”; “discharge.” In order to classify a standard as “met,” this had to be met for at least 90% of the clients.

## Results

### Socio-Demographic Characteristics of the Sample

Data on 109 CHR-P individuals were retrieved, 39 were excluded because discharge happened within the first 6 months. This resulted in a total of 70 individuals eligible to be included in this study. Sociodemographic characteristics are presented in **Table 2**. The mean age of the sample was 22.93 years (SD = 5.552, range 14–36), 30 individuals were females and 40 males. There were no significant differences in terms of age, gender, ethnicity across the four SLaM boroughs (see **Table 2**). Individuals were followed up for an average of 18.41 months (range 6–29). Employment and education status at baseline and at the last available follow-up are reported in **Tables 3** and **4**. Data on standards are reported below and summarized in **Table 5**.

**Table 2:** Socio-demographic characteristics of the sample.

	Total	SouthwarkN = 17	LambethN = 27	CroydonN = 12	LewishamN = 14	Statistics
Age	22.93SD 5.55	20.41SD 3.20	23.37SD 5.86	23.75SD 5.64	24.43SD 6.65	F = 1.70p = 0.17
Gender (F/M)	30/40	9/8	13/14	5/7	3/11	χ2 (3, N = 70) = 3.64p = 0.30
Ethnicity						
White	31 (44.3%)	7	11	5	8	χ2 (9, N = 70) = 15.93
Black	20 (28%)	3	11	2	4	p = 0.068
Asian	7 (10%)	0	3	2	2	
Other	12 (17.1%)	7	2	3	0	

**Table 3:** Employment status baseline—follow-up.

Employment status baseline	Employment status follow-up	
		Change status = 27
Full-time	Unemployed	2
Full-time	Part-time	1
Part-time	Sick leave	1
Part-time	Internship/volunteer	1
Student	Unemployed	4
Sick leave	Unemployed	1
Unemployed	Full-time	1
Unemployed	Part-time	1
Unemployed	Internship/volunteer	2
Sick leave	Full-time	1
Sick leave	Part-time	2
Student	Full-time	5
Student	Part-time	1
Student	Internship/volunteer	3
Unknown	Part-time	1
		
		**Same status = 40**
Full-time		10
Part-time		3
Student		11
Unemployed		16
		
		**Maternity = 2**
Part-time	Maternity	1
Unemployed	Maternity	1
		
		**Missing = 1**

**Table 4:** Education status baseline—follow-up.

Education status baseline	Education status follow-up	
		Progressed = 17
None	GCSE	5
GCSE	A levels	6
A levels	Started undergraduate degree	2
Started undergraduate degree	Completed undergraduate degree	4
		
		**Same level = 45**
None		5
GCSE		12
A levels		12
Started undergraduate degree		10
Completed undergraduate degree		5
Completed postgraduate degree		1
		
		**Missing = 8**

GCSE, General Certificate of Secondary Education.

**Table 5:** Number of standards met per client.

Standards met	Number of clients	%
**0**	**3**	**4,3**
**1**	**6**	**8,5**
**2**	**2**	**2,8**
**4**	**3**	**4,2**
**5**	**6**	**8,6**
**6**	**17**	**24,2**
**7**	**16**	**22,8**
**8**	**11**	**16**
**9**	**6**	**8,6**
	**70**	**100**

### Adherence to Standards

#### Standard 1: Quality of Vocational Assessment

Current vocational engagement (1.a.) was assessed at initial assessment for most clients (75%). All clients were assessed within the first 12 months. There were no significant differences across boroughs [χ2 (6, N= 70) = 3.241 p = .778]. Vocational goals were assessed (1.b) at initial assessment for 34.8% of CHR-P clients, and within 6 months for another 44.9% of clients. According to the records, vocational goals do not appear to have been assessed in 10% of cases. There were no significant differences across boroughs [χ2 (15, N = 69) = 20.849 p = .142]. With regards to assessment of support needs (1.c), these were assessed at initial assessment for 36.2% of clients, and within 6 months for another 34.8% of clients. According to the records, support needs do not appear to have been assessed for 18.8% of clients. There were no significant differences across boroughs (χ2 [15, N = 69) = 13.449 p = .568]. According to the records, vocational history (1.d) was recorded for 50% of included clients. There were no significant differences across boroughs [χ2 (3, N = 70) = 7.043 p = .071]. For standard 1, having at least 3/4 sub-standards met, was considered as overall “met”. For 56/70 clients (80%) standard 1 was met, therefore standard 1 was considered overall “not met” at the service level.

#### Standard 2: Vocational Activities Feature in Care Plan

The care plan was not retrievable through the electronic recording system for 42.9% of the included CHR-P clients (= 30). Out of all retrievable care plans (= 40), 75% had vocational activities recorded. There were no significant differences across boroughs [χ2 (6, N = 70) = 9.564 p = .144]. Standard 2 was met for 42.9% of clients (= 30), therefore standard 2 was overall “not met” at the service level.

#### Standard 3: Clients Have Access to a Vocational Support Program

Retrievable data suggests that only 20% (= 14) of included CHR-P individuals did not receive a specific intervention aimed at supporting employment or educational needs. Of the remaining (= 56), 62.9% of clients received support within the first 3 months, 22.2% within the first 6 months, and 17.3 within 12 months or shortly after. There were no significant differences across boroughs [χ2 (12, N = 68) = 10.331 p = .587]. Standard 3 was considered as “met” if clients received support within the first 12 months. 51/70 clients (72%) received support within the first 12 months, therefore standard 3 was considered “not met” at the service level.

#### Standard 4: Early Psychosis Services Liaise With Educational and Employment Providers

In 36% (= 25) of cases OASIS services liaised with vocational providers while in 37% (= 26) they did not. The remaining percentages are explained by clients not being employed or in education (21.4%), the vocational provider already being aware of clients’ difficulties (2.9%) or clients choosing to liaise with their employer/educational provider themselves (2.9%). There were no significant differences across boroughs [χ2 (12, N = 69) = 16.800 p = .157]. Standard 4 was considered as met if there was evidence that i) the service liaised with employer/education provider, ii) the employer/school/university was already aware,” or iii) the “client liaised with employer/school/university”. Standard 4 was met for 29/70 clients (42%), therefore this standard was overall “not met” at the service level.

#### Standard 5: Early Psychosis Services Liaise With Local Stakeholders

Stakeholders involved to support clients with their vocational needs varied greatly. They included parents or relatives, employment or support programs, educational institutions, and job centers. Borough-specific external support services were involved in 27.1% of cases, job centers were involved in 2.9% of cases, recovery colleges in 2.9% of cases, multiple stakeholders—including parents and relatives—in 11.4% of cases. For 41.4% (= 29) of clients there was no involvement of local or external stakeholders. The remaining was either referred to other clinicians or, in one case, to a solicitor. There were no significant differences across boroughs [χ2 (18, N = 70) = 18.982 p = .393]. Standard 5 was met for 41/70 clients (41.4%); therefore, it was considered as “not met” at the service level.

#### Standard 6: Support Is Focused Around Competitive Employment or Mainstream Education

Standard 6 is not specifically drawn from early psychosis guidelines although the NICE guidelines (34) on psychosis and schizophrenia in children and young people mention educational support aimed at mainstream education. This standard is a key principle of IPS (32) which recommends supporting people into mainstream employment and education as opposed to special programs or voluntary work. Out of all clients who did receive vocational support (56/70; 80%), this was always aimed at mainstream education and competitive employment. Standard 6 was met for all clients who received vocational support (100%), therefore it was considered as “met” at the service level.

#### Standard 7: Support Is Provided Based on People’s Interest

Out of all clients who did receive vocational support (56/70; 80%), this was always in line with their individual interests. Standard 7 was met for all clients who received vocational support (100%), therefore it was considered “met” at the service level.

#### Standard 8: Support Is Time Unlimited

Out of the clients who did receive vocational support (80%), in 62% of cases vocational support was terminated due to discharge, in 15% of cases vocational support was still ongoing at the time of data collection, and in 22.6% of cases vocational support ended with the termination of psychology sessions. There were no significant differences across borough [χ2 (9, N = 69) = 5.051 p = .830]. Standard 8 was considered as met if there was evidence that i) support was ongoing, or ii) support ended when the client was discharged from the service. Standard 8 was met for 42/70 clients (60%), therefore it was considered as “not met” at service level.

#### Standard 9: Benefits Counseling Is Provided

For 43% of included clients (= 30) this standard did not apply as clients were either financially supported by their parents, in full-time employment or not legally allowed to work and access benefits in the UK. Of the remaining, 50% (= 20) received benefits counseling and/or support in accessing financial means to support their return to education. There were no significant differences across borough [χ2 (9, N = 69) = 2.782 p = .972]. Standard 9 was considered as met if there was evidence that i) benefit counseling was provided or that ii) it was not applicable. Standard 9 was therefore met for 51/70 (72.8%) of clients, therefore it was considered as “not met” at the service level.

## Discussion

We sought to evaluate the provision of vocational interventions offered by OASIS, one of the oldest and largest EDP services in Europe and worldwide. We defined a set of nine standards based on the NICE guidelines and IPS principles and assessed whether current clinical practice in OASIS met these standards. Results showed that 80% of clients (56/70) met at least five out of nine standards (**Table 3**).

The results suggest that overall NICE standards of practice and the IPS principles were only partially met. In particular, standards 6 and 7 were met: results confirmed that the focus of the support provided is on helping clients to re-engage or remain in competitive employment or mainstream education and that this support is based on clients’ interests rather than providers’ judgment (32). This is particularly important as these standards were both based on IPS principles rather than on the NICE guidelines, suggesting that the OASIS team might already score highly on some of the IPS fidelity scale items (32).

Standards 1, 2, 3, 4, 5, 8, 9 were only partially met. OASIS appears to be doing particularly well with regards to assessing clients’ current vocational engagement (standard 1.a), vocational goals (standard 1.b), and support needs (standard 1.c). On the contrary, vocational history (standard 1.d) was formally assessed in only 50% of cases. This could be at least in part explained by the fact that 23.3% of clients were in mainstream education and 28% were working part or full-time, therefore assessing vocational history might not have appeared relevant at the time.

The assessment of standard 2, “vocational activities feature in care plan”, revealed that, among the retrievable care plans, vocational activities were recorded for the majority of clients (75%). Unfortunately, an electronic care plan was not retriable for 42% of cases, therefore it was difficult to assess adherence to this standard. It is possible that the care plans could have been saved in a paper format rather than electronically. In some cases, the care plan was identifiable within the clinical notes, it was however not included as part of this analysis as a formal care plan developed in collaboration with the client was not found.

Assessment of standard 3, revealed that clients had access to vocational support in 80% of cases. This is relatively high considering that OASIS does not yet have a dedicated vocational specialist. It is also encouraging that for the majority of clients (85%), when vocational support was offered, it was offered early on, within 6 months. On the other hand, the relatively high percentage of people who had access to vocational support could also be the result of the use of a broad definition which might not apply to specific vocational support program.

Standard 5 assessed whether OASIS was liaising with local or external stakeholders when providing vocational support. While there was variety in the type of stakeholders involved, for 41.4% of clients these were not involved at all. The external stakeholders included borough-specific external agencies (e.g. https://www.princes-trust.org.uk) or teams (such as recovery colleges, or vocational services) aimed at supporting young people or people with mental health issues in gaining employment. This is a valuable resource which however might not be equally present across all catchment areas within or outside the UK. However, it is important to remember that south London is one of the most deprived regions in the UK (38). Therefore, mental health services in south London are likely to experience increased difficulties in identifying suitable employment opportunities and people living in this area might experience greater difficulties in finding or keeping a job.

Standard 8, “support is time unlimited”, was also based on one of the IPS principles. When a client is taken on by the OASIS service, support and management is provided for up to 2 years, after which the person is discharged to a general practitioner or referred to another service, if appropriate. For 15% of the clients, support was still ongoing at the time of the audit. For 62% of clients, vocational support was terminated due to discharge suggesting that support was provided for as long as possible within the current structure of the team. For another 22.6% support ended with the termination of psychology sessions. Currently, OASIS offers up to 24 psychology sessions, which can be increased if needed. This is more than what is currently suggested as part of the manualized CBT treatment for those at risk of a first episode of psychosis (39) and indicates that although support ended with the psychology sessions, this has been provided over the course of several weeks. While the role of psychology is primarily to address psychological distress, within the current structure, psychology sessions also provided extensive vocational support. Vocational support provided might have not been delivered in a standardized way and might have been limited to discussion within the psychology sessions. A dedicated IPS worker would ensure the presence of dedicated time to work on vocational issues and engage in a more flexible way with external stakeholders (e.g. schools, employers).

Standard 9, “benefit counseling is provided” is one of the IPS principles (32). 43.5% of clients were employed, in education, supported by their parents or not legally allowed to access benefits in the UK, therefore this standard did not apply. Of the remaining, only 50% received advice on accessing benefits.

NICE guidelines recommend providing support with education and employment needs and goals; however, there is no specific recommendation as to which framework or theoretical model to use and how to integrate this within the current structure of the early detection and intervention teams (34, 35). In this context, IPS provides a clear framework in which different health care professionals can work conjunctly to help young individuals to complete their educational course successfully, to move toward employment and improve their mental wellbeing (23, 24, 27, 40, 41).

In the short-term, improving educational and employment support within the existing structure of the teams might require some adjustments. For example, the implementation of more standardized vocational assessments and recording of goals and needs. The fact that this was not done for all included clients might reflect the fact that in mental health services the main focus is on clinical symptoms, therefore, the support offered often takes the form of psychological intervention around clinical symptoms rather than vocational support. Nevertheless, this is an area that should be assessed routinely. In order to address this, we propose two changes. Firstly, the care coordinator could dedicate a specific session early in the care pathway to complete a more comprehensive and structured vocational assessment including vocational history. Secondly, automated prompts on the electronic care plan could be implemented to remind the care coordinator to include vocational goals and the support offered to work toward them.

In the long-term, EDP services like OASIS are likely to benefit from having an IPS worker whose task is solely to provide support with education and employment needs. This is particularly important given the current high rates of unemployment in this (i.e. 37% at the last available follow-up) and similar samples (4, 6, 8). The expectation is that having a dedicated IPS trained worker based in the team will improve performance on all standards and generally address the low social and occupational functioning (12). As the clients in EDP teams are relatively young (i.e. 14–35), they are likely to benefit from IPS with focus on both employment (26–28) and education (27, 29).

The results also showed that there are no significant differences in the nine standards across boroughs despite the fact that two of the four OASIS teams (i.e. Croydon and Lewisham) were set up only recently (i.e. 2014–2015). While this is reassuring samples were relatively small, therefore further analyses with larger samples are needed to confirm that there are no significant differences in the delivery of education and employment support across boroughs.

This study has a number of strengths. Firstly, OASIS is a well-established EDP service operating since 2001. The setting is therefore ideal to evaluate if NICE standards are met. Secondly, the case-load of the service made it possible to look at the different aspects of vocational interventions in a modest sample. This study has also a number of limitations. Firstly, results are limited to well-established and specialized team within SLaM NHS Foundation Trust. While this is one of the most deprived areas in the UK (38), we do not know to what extent these results can be generalizable to other areas in or outside the UK where employment, education, and training opportunities might differ. Thirdly, data was collected retrospectively, through the screening of electronic clinical records. This can be a limiting factor for at least two reasons. Data which is marked as missing, might have been present but not recorded electronically thus providing only a partial picture. Furthermore, data was initially recorded as part of routine clinical work, therefore, there was no formal quality check on how data was collected and entered. Despite this, we are confident the quality of the data is of satisfactory standard as periodic checks are carried out to ensure outcome measures are recorded consistently. Fourthly, data available did not allow to determine whether, to what degree and which element of vocational support influences health and vocational outcome. To address these limitations future studies should test feasibility, challenges, and benefits of implementing vocational interventions, such as IPS, in EDP teams in the UK. As prior studies indicated that difficulties during education or during employment contribute to increase distress (15, 42), future studies should also investigate the effect of implementing interventions that are specifically focused on improving coping strategies in this context (42, 43).

Much has been done to date to provide treatment as early as possible to young people who present with symptoms that suggest they might be at high risk of developing psychosis in the near future. However, this work suggests that there are areas that have not been addressed sufficiently and one of these is vocational recovery. Therefore, the focus of the intervention provided by EDP teams should go beyond management of symptoms and be broadened to include interventions that selectively target vocational recovery. In this context, IPS offers an implementable framework which is expected to enhance the work of EDP teams and help young people with their vocational goals.

## Ethics Statement

This study was carried out as part of a clinical audit in accordance with the recommendations of South London and Maudsley Psychosis Clinical Academic Group.

## Author Contributions

ST designed the evaluation, prepared the first draft of the manuscript, and assisted with data extraction and data analysis. LG assisted with the design of the evaluation, contributed to prepare the first draft of the manuscript, and performed the data extraction. SV performed the data extraction and data analysis. TS designed the evaluation and assisted with data extraction and data analysis. All co-authors contributed to the critical revision of the manuscript.

## Funding

ST is supported by a Brain and Behavior Young Investigator award (NARSAD YI, 24786) and by a Maudsley Charity Grant (1510).

## Conflict of Interest

The authors declare that the research was conducted in the absence of any commercial or financial relationships that could be construed as a potential conflict of interest.
